# Correspondence

**Published:** 1888-08

**Authors:** G. E. Corbin

**Affiliations:** St. Johns, Mich.


### CORRESPONDENCE.

### Disfigured for Life.

St. Johns, Mich., July 23, 1888.

Dr. Taft, Dear Sir:

Permit me to call attention to an unfortunate case recently falling under my notice. It illustrates in a striking manner the great damage being done all over the country by injudicious, unnecessary, wholesale extraction of human teeth. A Miss of sixteen years of age recently consulted me, who, four years ago fell upon ice, and broke off her left superior central incisor, near the alveolar process. Physicians, and a dentist in a neighboring town, where she resided were consulted, and as the result of the consultation, the root, though sound and firm, was extracted. The changes of four years time, at that age, have been great, and unfortunate. The right superior central and lateral incisor now slant diagonally across her mouth to the left at an angle of nearly forty-five degrees. The left superior canine and lateral incisor have advanced forward, and the left superior bicuspids have fallen inward so that their external cusps shut inside of the left lower bicuspid teeth. The girl and her mother both insist, that prior to the accident her teeth all articulated in the normal position. This great disfigurement seems to have been the result of the improper extraction of a single tooth. The physicians consulted will compare favorably in professional intelligence with their brethren in small country towns generally. The physicians simply argued that the root was worthless, and its prompt extraction would relieve severe suffering. That much they could do, and little more than that has been attempted by physicians, as such, in centuries. To the credit of the dentist let is be said that "he hesitated" before extracting.

Doubtless he was conscientious, but certain it is, he was not fully up to the standard of the times. As it was, the "keystone" to the arch was lost, and the symmetry, strength, and beauty of the arch necessarily destroyed. All dentists *should* know, and many intelligent members of the general community certainly now *do* know that the root of the broken tooth above cited could have been restored to health and comfort and supplied with a strong durable crown that would have defied detection at ordinary speaking distance.

A very small percentage of the general community, however, have any just conception of the nature or extent of the injury sustained by the extraction of such roots. *Some* teeth must be lost. Most of those that are lost, might be saved. Ignorance destroys teeth. Intelligence preserves them. This is true on the part of all concerned—dentists, physicians and patients.

G. E. CORBIN.

